# Acetate of Iron an Antidote to Arsenical Preparations

**Published:** 1846-05-01

**Authors:** M. Duplas


*Acetate of Iron, an antidote to Arsenical preparations.*—It appears from experiment that thehydrated per-oxide of iron possesses no efficacy as an antidote, when arseniate of potash (Fowler's solution) has been employed; it only acting when the uncombined acids have been used. The author recommends as a substitute in this case, the per-acetate of iron prepared in the ordinary way—(there should be an excess of the per-oxide). This preparation is administered largely diluted with water, it being thus much more efficacious.

M. Duplas, Phil. Mag. *From American Jour. of Science and Arts.*

